# A Home-Based, Music-Cued Movement Program Is Feasible and May Improve Gait in Progressive Supranuclear Palsy

**DOI:** 10.3389/fneur.2019.00116

**Published:** 2019-02-19

**Authors:** Joanne E. Wittwer, Margaret Winbolt, Meg E. Morris

**Affiliations:** ^1^Physiotherapy Discipline, La Trobe Centre for Sport and Exercise Medicine Research, School of Allied Health, La Trobe University, Melbourne, VIC, Australia; ^2^Australian Institute for Primary Care and Ageing, La Trobe University, Melbourne, VIC, Australia; ^3^Healthscope and La Trobe Centre for Sport and Exercise Medicine Research, School of Allied Health, La Trobe University, Melbourne, VIC, Australia

**Keywords:** acoustic simulation, supranuclear palsy—progressive, walking speed, neurological rehabilitation, gait rehabilitation

## Abstract

**Objectives:** To understand the benefits and feasibility of using supervised, home-based, music-cued training to improve gait speed and stability in community-dwelling people with Progressive Supranuclear Palsy.

**Design:** Feasibility trial incorporating a single group repeated-measures design.

**Setting:** Human movement laboratory and participants' homes.

**Interventions:**Two training sessions per week, conducted by experienced physiotherapists over 4 weeks. Each home training session consisted of a range of activities in standing or walking, with, and without auditory cues. Rhythmic auditory cues were played via a portable digital music player and consisted of metronome beats and individually chosen, commercially available rhythmic music tracks.

**Main Outcome Measures:** Spatiotemporal gait measures were recorded using an 8 m long GAITRite® mat. Participants walked without cues at self-selected comfortable pace. The Progressive Supranuclear Palsy and Unified Parkinson's Disease Rating Scales were administered at baseline. Addenbrooke's Cognitive Examination-III, Geriatric Depression Scale, Assessment of Personal Music Preference Scale, and Physiological Profile Assessment were administered at baseline and retest.

**Results:** At baseline, two of the five community-dwelling participants with Progressive Supranuclear Palsy walked with normal speed and low gait variability. Of the remainder who walked with slower, more variable patterns, two walked faster at retest, one by a clinically meaningful amount. Four participants reduced their timing variability at retest and three reduced step length variability. All participants reported high satisfaction levels with the program.

**Conclusions:** When delivered at home with the support of caregivers, music-cued gait training can provide a feasible approach to improving disorders of gait stability in people with this rare, degenerative condition. Movement to music is engaging and enjoyable which can facilitate adherence to therapy.

**Clinical Trial Registration :** ANZCTR 12616000851460. http://www.anzctr.org.au/

## Introduction

Progressive Supranuclear Palsy (PSP) is a debilitating and rare disease (1, 2). It has a reported prevalence of only five per 100,000 (3) or about 380,000 people worldwide, despite being the most frequently occurring form of atypical parkinsonism (1). It is commonly misdiagnosed as Parkinson's disease (PD) so this may be a conservative estimate (3). It is associated with early decline in gait, balance, and movement speed (4). The cause of PSP is not known, hence treatment is currently symptomatic (5). People living with PSP are generally referred for rehabilitation with the aim of helping them to walk more easily and to prevent falls which are a hallmark of the disease (5), however evidence to support rehabilitation strategies is scant (6). Costs of care are significantly associated with symptom severity so interventions to improve mobility have the potential to reduce these costs (7).

The effects of physiotherapy, including exercise, and task-based training for people with PSP have not been robustly investigated despite clinical reports of benefit (6) together with findings that their functional disability is mostly due to physical, and not cognitive or psychiatric problems (8). The only randomized controlled trial (RCT) of rehabilitation for PSP reported improvement in gait, balance, and number of falls compared to baseline for both groups using an intensive, aerobic, goal-based intervention including treadmill training in an inpatient setting (9). Exercise studies in animals (10–12) and for people living with Parkinson's disease (PD) (13–15) reported a slowing in the rate and level of basal ganglia (BG) disease progression with intensive exercise. There may be potential for exercise to modify the trajectory of PSP disease progression over time, although this has not yet been verified with RCTs.

One of the challenges faced by current clinicians is knowing which modes of exercise and gait rehabilitation to use. PSP, PD, and Alzheimer's disease (AD) share some common pathophysiology and movement disorders so clinicians sometimes use PD or AD rehabilitation strategies to manage movement problems associated with PSP. Whether this is valid remains open to question. Despite similarities in different parkinsonian disorders, differences such as the unique balance problems seen in PSP (16) mean that treatment approaches need to be tailored for different disorders.

Gait training using rhythmic music or simple beat cues is a form of physical therapy that holds promise. In humans, perception of rhythm is a strong trait that does not need to be trained (17). Areas of the brain that control rhythm processing are closely related to those involved in movement and include premotor cortex, supplementary motor area (SMA), cerebellum, and BG especially the putamen. In PD the putamen is reported to be one of the most affected neural structures so it has been suggested that external music cues may entrain rhythmic gait by replacing the putamen's faulty automatic internal clock (17). Cues may also either facilitate or bypass the impaired BG-SMA pathway (17). There are reported differences in the effects of different types of auditory cues (18) with “groove” music suggested to drive the motor system (19) and simple beat cues producing effects over a longer period in people with degenerative BG disease (20).

In AD pathology which does not feature the lack of internal beat generation associated with BG dysfunction, music cued-exercises have also been shown to improve motor performance (21, 22). A suggested mechanism is that external cues may induce motor learning rather than just replacing faulty timekeeping (23). Music can also influence emotions which in turn affect movement. Rhythms judged subjectively as “beautiful” have been shown to enhance ventral premotor cortex activity more than non-preferred rhythms (24).

Increased gait variability is associated with increased falls risk in healthy older people (25) and those with AD (26). Therapy using rhythmic music aimed at reducing gait variability has previously been shown to be effective in people with AD and PD (21, 27–29). It is therefore plausible that actively entraining continuous gait cycles to a rhythmic beat could not only improve gait speed but reduce gait variability in people with PSP. Spontaneous foot and finger tapping to musical stimuli has been reported in people with PSP, despite an inability to perform the same movements on request (30).

Living with a severe degenerative disease can restrict access to services such as community-based therapy sessions due to factors such as the regular need to rely on others for transport. To date, PSP studies have been conducted in laboratory or clinical settings. Home-based gait rehabilitation for people living with PSP has not previously been investigated.

The aim of this study was to evaluate the feasibility and effects of using rhythmic auditory cues to improve the walking patterns of community-dwelling people with PSP. In this study we used rhythmic music and simple beat cues to combine the different enhancing effects of each type of cue. As well as examining whether it was practical and feasible to deliver this intervention in the home setting, we examined whether gait variability, amplitude and timing improved with a supervised home program of music and metronome cued movements.

## Materials and Methods

### Participants

Participants were recruited from neurologists in the Melbourne metropolitan region, Australia. Invitations to participate in this study required a diagnosis of probable PSP made by a specialist neurologist according to consensus criteria (31). Other inclusion criteria were the ability to walk 100 m independently without a gait aid, adequate hearing to conduct a conversation (including with hearing aids), and the capacity to comprehend training program instructions. Exclusion criteria included limited cardiac or pulmonary capacity or a diagnosis with any other neurologic or musculoskeletal co-morbidity or pain significantly affecting gait. Participants required clearance from their local doctor to perform exercise of mild to moderate intensity. Ethical approval for the study was granted by the university ethics committee (HEC16-016) and all participants gave informed consent.

### Assessments

Participants attended the university movement laboratory for assessment immediately before (baseline) and after (retest) the 4 week home-based training sessions. Relevant personal characteristics were recorded during the baseline session. During each of the assessment sessions participants completed the Addenbrooke's Cognitive Examination-III (ACE-III) (32) which assesses five cognitive domains (attention/orientation, memory, verbal fluency, language, and visuospatial abilities); the Geriatric Depression Scale (GDS) (33); the Assessment of Personal Music Preference Scale (34), the Physiological Profile Assessment (35). The Progressive Supranuclear Palsy Rating Scale (PSPRS) (36) and the Unified Parkinson's Disease Rating Scale (UPDRS) (37) were scored only during baseline assessments.

Gait spatiotemporal measures were recorded using an 8 m long GAITRite® mat (CIR Systems, Inc., 12 Cork Hill Rd, Franklin, NJ 07416, USA). Measurements using this system have previously documented high reliability when combining >30 steps for healthy older groups and those with PD and AD (38, 39). Participants walked on the mat at self-selected comfortable pace (“As if you were walking to the local shop”). Two familiarization walks were performed followed by a further six walks. Walks were commenced and finished 2 m beyond each end of the mat to remove acceleration and deceleration phases. Short rests between walks were taken as required. Walks were repeated if the participant spoke, became obviously distracted or strayed off the mat.

A specifically designed questionnaire evaluating program organization and content on a five point Likert scale was completed by participants. It was filled in at home and returned via reply-paid post.

### Intervention

Each of the eight physiotherapy sessions was conducted in the homes of participants. Two training sessions per week, each of up to 1-h duration were scheduled over 4 weeks and were conducted by experienced registered physiotherapists. As the overall weekly amount of practice or “dose” of the intervention is the main determinant of motor learning, specific intervals were not specified between sessions in each week to maximize convenience for participants and care-givers. The training protocol is outlined in Table 1. It was based on a previously reported program used with patients with stroke (40) and was designed to be sufficiently flexible to accommodate different levels of gait function. It was progressively modified each week to include different and more challenging cued gait tasks and tailored for each participant. Each training session commenced with seated warm-up activities followed by a range of different activities in standing or walking with varied cue types and tempi (Table 1).

**Table 1:** Details of gait training program activities and rhythmic auditory cues (RACs).

**TRAINING PROGRAM ACTIVITIES**	
Warm-up (Seated):	Seated toe/heel tapping/knee lifts/hand clapping
Upright Activities (Stepping):	- Forwards/Side/Back Stepping - Crossover steps - Step ups - Sequences of stepping - Add arm movements
Walking:	- Walk on the spot - Straight line walking forwards/backwards/sideways - Walking with turns forwards/backwards/sideways - Walk stop and go - Walk with turns /stop and go - Add arm movements
Vocalizing:	- Singing “la la”/song lyrics; counting “1, 2”
Cue Fading:	- Maintain tempo of rhythmic activity as cue is muted and then made audible again.
**RHYTHMIC AUDITORY CUES (RACs)**	
RAC Type:	- Metronome software application for handheld digital music player (Pro Metronome® http://eumlab.com/pro-metronome/) - Rhythmic Music—a variety of commercially available music files
RAC Player:	- All cues played via a handheld digital music player (Apple Ipod®) connected via WIFI® to a portable speaker (Ultimate Ears®).
Music Selection Criteria:	- An unchanging tempo with a clearly discernible rhythm in 2/4 or 4/4 meter - Music pieces in a range of styles (classical, popular and marches) were varied through the sessions - Participants nominated preferred selections to be used during each session.
RAC Tempi:	- Commercial software (Tempo Magic Pro®) was used to manipulate music cue tempo without altering pitch. - Comfortable pace walking cadence - 110% comfortable pace walking cadence - 90% comfortable pace walking cadence

*Two home visit sessions per week for 4 weeks with individual session duration of no more than 60 min. Training program activities and RAC type and tempo varied in each session according to participant ability and progress*.

The mean self-selected comfortable speed cadence from the baseline testing session was used as the comfortable pace tempo for cues during the first week of training and then increased or decreased according to participant ability with each activity.

During the first physiotherapy intervention session, each participant was given a small personal digital music player and headphones (SanDisk Clip Sport® Western Digital Technologies, Inc., 951 SanDisk Drive, Milpitas, CA 95035-7933, USA) pre-loaded with rhythmic music. Participants were asked to listen to this music while seated for at least 15 min each day. Music was chosen according to personal preference using a standardized questionnaire (34) and ranged from classical and military marches to popular music. Participants were asked to focus on the rhythm of the music pieces and then move their arms and/or tap their toes to the beat. Practice sessions were recorded in a diary.

### Data Analysis

Gait data were processed according to a previously described method (39). In summary, for each participant the velocities of individual walks were compared. The “outlier” walks (i.e., walks with velocity of more than 10 cm/s different from the median velocity for all walks for each individual participant) were removed and remaining walks were then combined for each condition. For each participant stride numbers were matched for baseline and retest tests. The gait measures calculated were spatiotemporal measures of velocity, stride length and stride time; and variability measures of stride time and stride length (using the coefficient of variation).

## Results

Five community-dwelling people with PSP were recruited over an 8 month period in Melbourne, Australia. Three were from the private caseload of a neurologist specializing in PSP management and two from a community physiotherapy program. All participants were able to complete the eight intervention sessions and no adverse events were reported. Measurement score ranges are presented where reporting individual values may compromise participant confidentiality. The rarity of PSP necessarily limited the scope for recruiting participants for this feasibility study, and therefore the ability to meet the criteria for CONSORT reporting has also been limited.

Participants were 3 females and 2 males with an age range of 54–74 years. Time since diagnosis ranged from 1.1 to 12.8 years with a median of 4.7 years. BMI scores ranged from 20 to 38 kg/m^2^ with two participants classed as normal weight and two as pre-obesity (41). Number of prescription medications ranged from 1 to 4. Vision scores using the Melbourne Edge test for measuring contrast sensitivity were within age-matched value ranges for all but one participant. Number of falls in the previous 12 months ranged from 2 to 20 with a median value of 12. The participant who had fallen twice in the previous year was very cautious when walking due to a previous injurious fall. Cognitive screening results using the ACE-III indicated that only one participant (Participant 1—see Table 2) may have dementia. Results for the verbal fluency tests which rely on executive function were similarly low for that participant. All participants lived in their own homes except one who lived in supported accommodation.

**Table 2:** Participant characteristics.

**Participant Characteristic**	**Participant 1**	**Participant 2**	**Participant 3**	**Participant 4**	**Participant 5**
Number of falls in previous 12/12 (retrospective)	20	2	5	12	12
PSPRS score (total score 100^#^)	41	25	30	32	29
PSPRS Gait subscale (total score 20)	11	10	9	6	10
MDS UPDRS (total score 260^+^)	87	93	85	73	66
MDS UPDRS Motor Examination subscale (total score 132)	54	62	42	42	31
	**Baseline**	**Retest**	**Baseline**	**Retest**	**Baseline**	**Retest**	**Baseline**	**Retest**	**Baseline**	**Retest**
ACE III score (total score 100^*^)	79	77	94	89	97	99	86	89	91	97
ACE III Fluency score (total score 14[Table-fn TN4])	5	3	10	10	12	14	9	10	8	13
GDS (total score 15^∧)^	7	7	5	4	7	10	1	1	13	12
Quadriceps Strength (Age-matched normative range) (kg)	24	28 (33-61)	19	13 (18-35)	10	22 (24-44)	31	32 (31-59)	6	12 (18-35)
Satisfaction Survey (max score 55)	–	53	–	51	–	52	–	48	–	45

# Progressive Supranuclear Palsy Rating Scale−0 is normal;

+ Movement Disorders Society Unified Parkinson's Disease Rating Scale−0 is normal;

* Addenbrooke's Cognitive Examination—III: 2 cut-offs for suspicion of dementia 88 (100% sensitive, 96% specific) and 82 (93% sensitive, 100% specific);

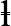
Fluency domain relies heavily on executive function;

∧ *Total Score >5 points is suggestive of depression. Scores >10 almost always indicate depression*.

Given the wide variation in their movement disorders and stages of disease progression the individual results for each participant are presented (Tables 2, 3).

**Table 3:** Change in gait measures from baseline to retest—self-selected comfortable pace walking.

	**Velocity (cm/s)**		**Stride time (s)**		**Stride time variability (CV %)**		**Stride length (cm)**		**Stride length variability (CV %)**	
**Participant number**	**Baseline**	**Retest**	**Baseline**	**Retest**	**Baseline**	**Retest**	**Baseline**	**Retest**	**Baseline**	**Retest**
P1	73.7	66.7	1.22	1.25	6.24	**4.17**	89.80	83.30	5.87	**4.84**
P2	78.2	91.3^*^	1.08	**0.98**	2.66	2.92	84.69	**89.77**	4.56	3.57
P3	122.4	119.3	1.13	**1.12**	2.70	**2.00**	138.25	133.20	1.38	2.00
P4	155.3	**159.6**	0.96	**0.91**	2.52	**2.44**	149.24	145.16	2.53	2.69
P5	88.7	**89.6**	1.13	1.15	3.56	**2.84**	99.73	**103.28**	4.11	**2.93**

* *Improved values at retest (bold); Improved value of clinical significance i.e., >11 cm/s (42)*.

Participant 1 mostly used a single point stick to walk indoors, however was able to complete all test trials with no gait aid and close supervision. Participant 1 was the most severely physically affected of the group, with slow, and variable walking. Participant 1 reported fluctuating motor function from day to day.

Participant 2 walked with supervision and was cautious when walking following a previous injurious fall. At baseline multiple episodes of freezing of gait were evident. The increase in velocity at retest corresponds to a “large” change in UPDRS Motor Scale scores (43) although stride length was still well below age-matched values (44). Participant 2 was the best “responder” to the cued training. That is, walking speed improved by a clinically meaningful amount (i.e., >11 cm/s) (42) at retest combined with improvements in three of the other four measures. Participant 2 reported using a strategy of singing some of the songs used as music cues when walking as this made walking feel easier (45).

Participant 3 enjoyed the program with a satisfaction score of 52/55, especially training with self-selected music choices.

Participant 4 regularly walked long distances in the local neighborhood for exercise, despite experiencing frequent episodes of blepharospasm, scoring lowest on the PSP gait subscale, and falling on average once per month. Participant 4 was however, the only participant whose knee extension strength was within age-matched normal range (35).

Participant 5 used a 4-wheeled frame but was able to walk without a gait aid at slower speed. Participant 5 reported distress with current life circumstances although also reported feeling more motivated to exercise as a result of participating in the music cued movement training.

### Group Baseline Performance

Despite the shared diagnosis, there was considerable variation across the five participants in motor presentation. Only one of the five participants (Participant 3) walked at a comfortable pace within age-matched normative values with Participants 1, 2, and 5 all walking more slowly than 1 m/s [a cut-off value for increased risk of functional limitations (46)]. Participant 4 walked more quickly than normative values however may have been walking at greater than self-selected comfortable pace due to the feeling of being “tested” during the gait assessments. Spatial variability was higher than published normative values for two out of five participants and timing variability exceeded published norms for one participant (44). Only one of the five participants recorded a knee extensor strength value within age-matched published norms at baseline (35), despite two of the participants (Participants 3 and 4) walking at speeds within normal range. Four of the five participants had GDS scores suggestive of depression (33). The participant with the lowest GDS score was also the participant who was most independent in terms of community and social participation.

### Group Retest Performance

All of the participants enjoyed the intervention sessions and the independent practice task (moving in time to music), with most reporting they listened longer than 15 min and would continue with this activity after the trial. GDS scores were however largely unchanged. The home-based intervention sessions were reported to be easy to fit into participants' schedules and reduced the need for caregiver involvement. Participant 2 increased comfortable pace velocity by a clinically meaningful amount following the intervention sessions, however did not reach 1 m/s. No participant improved in all five of the post-intervention measures however three increased “comfortable pace” walking speed following the intervention. Timing variability reduced for four of five participants and spatial variability in three of five. This effect of temporal cues on both temporal and spatial gait measures has also been reported in a group with AD (21). Four participants improved knee extensor strength, one by 10 kg (Participant 3), although only one participant's (Participant 4) strength was within normal values.

## Discussion

Although gait disorders are a debilitating consequence of PSP, this is one of the first reports of gait rehabilitation for people living with this rare neurological condition. In many respects, the walking patterns of people with PSP tested in this trial resembled those of people with idiopathic PD who have short and slow footsteps (47, 48) and reduced ground clearance.(49) As with PD and AD, the people with PSP in this trial mostly responded positively to the rhythmic music and metronome auditory cues. In particular, gait timing variability which is associated with increased falls risk (25, 26) improved (reduced) with music-cued exercises however this needs to be verified with controlled research. Gait variability measures have not previously been reported for people with PSP and no published studies have reported the effects of any type of intervention on gait variability in people with this condition.

The reasons why auditory cues improve gait in PSP may relate to the pathogenesis of this BG disorder. Like PD, the BG pathways are affected in PSP and there is extensive subcortical neurofibrillary degeneration mostly in the Globus pallidus, subthalamic nucleus, substantia nigra, and cerebellar dentate nucleus (50, 51). This means that gait, balance, and sequential movements are compromised and community ambulation can be curtailed (52). The neural mechanisms underlying postural stability are not fully understood, however the pontomesencephalic region has been shown in cats to contribute to the control of the accompanying postural adjustments during voluntary gait modifications (53). In people with PSP, the high neuronal loss observed in the pontomesencephalic region could therefore contribute to the high incidence of gait problems and falls (53).

It is of concern that despite the ability to improve knee extensor weakness demonstrated by most of the trial participants in response to the music-cued intervention, people in this group were not routinely engaging in sufficient exercise to maintain adequate strength essential for optimizing physical independence (54). Some of the participants were undertaking other exercise activities however it seems likely that they were not engaging in sufficiently challenging activities. For example, well established guidelines for physical activity for older adults recommend progressive weight training on at least 2 days/week and daily endurance activities at an intensity rated between “moderate” and “vigorous” (55). For those with chronic health conditions exercise should still be done but should be modified to be “as tolerated” (55). Motivational music has been shown to improve endurance during treadmill walking with participants taking longer to reach fatigue than “neutral” music or white noise so music-cued exercise may be helpful in assisting people with PSP to achieve a higher exercise dosage or intensity (56).

There were some limitations of this trial. The rare nature of this condition and the time constraints of the project limited the size of the sample. Although there were positive findings for music-cued gait training, the results were not uniform. For the purposes of standardization of gait measurement we omitted walks where there was obvious “dual-task” interference. This limits generalization of study findings to walking in situations where attention may need to be divided. We chose not to exclude people with psychiatric disorders and the symptoms of depressive illness reported in four participants, even though these have been associated with reduced gait speed and stride length (57, 58) and also increased gait variability (58). Given the nature of PSP it could be reasonably expected that mood would be affected to varying degrees in most individuals living with this disorder. The PSPRS and UPDRS were scored at baseline to characterize participants but were not scored at follow-up as they have limited sensitivity compared to the predicted magnitude of change and are time-consuming and tiring for this group.

Despite these limitations, all participants were positive about engaging in an enjoyable physical activity especially with music tailored to their preferences, and would have continued the intervention if possible. Caregivers, who are likely to experience substantial burden of care (59), were also positive about the convenience of the home-based sessions, the opportunity to engage in safe physical activity and the effects on mood.

## Conclusion

An eight session home-based program of music cued gait training was feasible for people living with mild to moderately severe PSP and was associated with improvements including reduced variability in temporal and spatial measures of walking. These results should be verified in a controlled trial together with an economic evaluation of the program. The extent to which other physical therapies such as strength training, aerobic activities, “pole walking,” dancing, and visual cueing are helpful, also awaits exploration.

## Author Contributions

JW was involved in the conception and design of the study, data acquisition, analysis and interpretation, and the drafting and critical revision of the article. MW was involved in the conception and design of the study, and critical revision of the article. MM was involved in the conception and design of the study, data analysis and interpretation, and the drafting and critical revision of the article.

### Conflict of Interest Statement

The authors declare that the research was conducted in the absence of any commercial or financial relationships that could be construed as a potential conflict of interest.
